# Electrical Transport Interplay with Charge Density Waves, Magnetization, and Disorder Tuned by 2D van der Waals Interface Modification via Elemental Intercalation and Substitution in ZrTe_3_, 2H-TaS_2_, and Cr_2_Si_2_Te_6_ Crystals

**DOI:** 10.3390/nano15100737

**Published:** 2025-05-14

**Authors:** Xiao Tong, Yu Liu, Xiangde Zhu, Hechang Lei, Cedomir Petrovic

**Affiliations:** 1 Center for Functional Nanomaterials, Brookhaven National Laboratory, Upton, NY 11973, USA; 2 Condensed Matter Physics and Materials Science Department, Brookhaven National Laboratory, Upton, NY 11973, USA; 3 Materials Science and Chemical Engineering Department, Stony Brook University, Stony Brook, NY 11790, USA

**Keywords:** electrical transport, electronic structure, 2D van der Waals interfaces, coherent electronic phases, intercalation

## Abstract

Electrical transport in 2D materials exhibits unique behaviors due to reduced dimensionality, broken symmetries, and quantum confinement. It serves as both a sensitive probe for the emergence of coherent electronic phases and a tool to actively manipulate many-body correlated states. Exploring their interplay and interdependence is crucial but remains underexplored. This review integratively cross-examines the atomic and electronic structures and transport properties of van der Waals-layered crystals ZrTe_3_, 2H-TaS_2_, and Cr_2_Si_2_Te_6_, providing a comprehensive understanding and uncovering new discoveries and insights. A common observation from these crystals is that modifying the atomic and electronic interface structures of 2D van der Waals interfaces using heteroatoms significantly influences the emergence and stability of coherent phases, as well as phase-sensitive transport responses. In ZrTe_3_, substitution and intercalation with Se, Hf, Cu, or Ni at the 2D vdW interface alter phonon–electron coupling, valence states, and the quasi-1D interface Fermi band, affecting the onset of CDW and SC, manifested as resistance upturns and zero-resistance states. We conclude here that these phenomena originate from dopant-induced variations in the lattice spacing of the quasi-1D Te chains of the 2D vdW interface, and propose an unconventional superconducting mechanism driven by valence fluctuations at the van Hove singularity, arising from quasi-1D lattice vibrations. Short-range in-plane electronic heterostructures at the vdW interface of Cr_2_Si_2_Te_6_ result in a narrowed band gap. The sharp increase in in-plane resistance is found to be linked to the emergence and development of out-of-plane ferromagnetism. The insertion of 2D magnetic layers such as Mn, Fe, and Co into the vdW gap of 2H-TaS_2_ induces anisotropic magnetism and associated transport responses to magnetic transitions. Overall, 2D vdW interface modification offers control over collective electronic behavior, transport properties, and their interplays, advancing fundamental science and nanoelectronic devices.

## 1. Introduction

The discovery of graphene in 2004 revolutionized materials science, sparking extensive research into two-dimensional (2D) materials. These materials, such as graphene, transition metal dichalcogenides (TMDs), and other van der Waals (vdW) materials, are bonded through weak vdW forces, allowing them to be exfoliated into atomically thin sheets [1,2,3] with remarkable and exceptional electronic, optical, mechanical, and thermal characteristics [2,4,5,6,7]. The study of electrical transport properties in 2D vdW materials is driven by both fundamental scientific interest and potential practical applications. From a basic science research perspective, 2D materials provide a unique opportunity to explore how reduced dimensionality, quantum confinement, and symmetry breaking lead to novel electrical transport behaviors distinct from their bulk counterparts. In 2D systems, electronic properties are often governed by novel phenomena such as massless Dirac fermions [4], enhanced collective electron behavior and electron–electron correlations [8], topologically protected electronic edge states [9], and spin–valley degrees of freedom [10], all of which enable the investigation of novel transport mechanisms [11,12]. Moreover, the band structure of 2D materials is highly tunable through external factors like strain, external electric or magnetic fields, chemical doping, and the stacking and/or orientational twisting of multiple layers [13,14,15,16,17,18,19]. This enables researchers to identify key parameters and external stimuli that govern and optimize the desired electrical transport properties [11]. This tunability makes them attractive for the exploration of a wide range of phenomena, including topological states, spin-polarized transport, valley-dependent electronic transport, superconductivity (SC), and other emergent quantum phases [16,17,18,20,21,22,23], providing a rich platform for exploring novel transport behaviors and new physics in condensed matter physics. Beyond fundamental research, these novel electrical transport properties hold promise for innovative applications in advancing next-generation nanoelectronics, optoelectronics, spintronics, and valleytronics, enabling smaller, faster, more energy-efficient, and flexible technologies in fields such as environmental science, clean energy, quantum computing, and artificial intelligence [24,25,26,27,28,29,30,31].

Significant achievements in the study of electrical transport in 2D vdW materials have expanded our understanding of both fundamental physics and potential technological applications. Graphene, as the first well-known 2D material, has revealed exceptional electrical transport properties. Charge carriers in graphene behave as massless Dirac fermions, with a linear energy dispersion dependent on momentum, resulting in high mobility and ballistic transport, even at room temperature [4]. Topologically protected edge states result in dissipationless transport, immunity to backscattering, and robust spin-polarized edge currents [9,11]. Edge disorder in narrower ribbons of graphene can cause Anderson localization, trapping electron wavefunctions and creating a conduction gap that blocks current flow, which disrupts graphene’s typical ballistic transport [32]. The quantum Hall effect can emerge in graphene, highlighting the significance of reduced dimensionality and the interplay of relativistic charge carriers with external magnetic fields in governing transport properties [33]. These features make graphene ideal for advanced electronic and spintronic applications.

Monolayer MoS_2_, a TMD, exhibits unique electrical transport properties due to broken inversion symmetry, which induces intrinsic out-of-plane polarization between the molybdenum and sulfur layers. This polarization enhances spin–orbit coupling and enables spin–valley coupling [21], modifying carrier mobility and enabling valley-polarized charge transport, such as the valley Hall effect [34,35]. The interplay between spin and valley degrees of freedom, along with the reduced dimensionality, facilitates valley-dependent transport and spin-polarized currents, making MoS_2_ ideal for spintronic and valleytronic applications in quantum computing and optical sensing [21].

WTe_2_, a 2D type-II Weyl semimetal, exhibits distinctive electrical transport properties due to the presence of Weyl fermions, which are massless particles with chiral charge carriers resulting from broken inversion symmetry [22,36]. These fermions are characterized by their momentum and spin being locked together, leading to unique transport behaviors. The strong spin–orbit coupling in WTe_2_ enhances the Berry curvature, which acts like a magnetic field for the electrons, contributing to the anomalous Hall effect. This results in topologically protected edge states and dissipationless transport along certain directions. The chiral anomaly, in which the electric and magnetic fields interact to produce nonlinear responses, further facilitates these unique transport behaviors. The combination of these features gives WTe_2_ high mobility, making it ideal for spintronic and quantum devices that require robust, topologically protected states essential for error-resistant quantum computing.

In hybrid 2D materials formed by coupling a topological insulator with a superconductor, the proximity effect induces a topologically non-trivial superconducting phase in the topological insulator. In this phase, topologically protected gapless edge states, arising from broken translational symmetry, close the superconducting gap at the edges or boundaries, leading to the formation of Majorana zero modes—quasiparticles that are their own antiparticles and follow non-Abelian statistics. These Majorana zero modes can be identified by zero-bias conductance peaks in electrical transport measurements [37]. The robustness of these gapless edge states against local perturbations makes the topologically protected Majorana zero modes promising candidates for quantum information computing, especially in the development of fault-tolerant qubits [23,37,38,39,40,41,42].

Phosphorene, a single or few atomic layers of black phosphorus, exhibits unique electronic and transport properties, including high hole mobility, anisotropic transport, a high on/off ratio in field-effect transistors (FETs), and tunable electronic characteristics due to its orthorhombic crystal structure and anisotropic band structure [43,44,45]. The high hole mobility arises from a flat valence band, reducing scattering, while weak electron–phonon interactions further enhance mobility by minimizing carrier–lattice scattering [45,46]. The transport properties of phosphorene are highly anisotropic, with higher mobility along the armchair direction due to stronger orbital overlap, and lower mobility along the zigzag direction due to weaker bonding. The direct bandgap, which can be modulated by layer number or strain, enables efficient on/off switching in FETs [44].

Controlled stacking and twisting of the same or different 2D vdW materials into heterostructures or superlattices enables the tuning of the assembly’s electronic properties, including interlayer coupling, band gaps, Fermi surface characteristics, and charge transport [16,17,19,47]. For instance, small magic-angle twisted-bilayer graphene, or WSe2, can dramatically alter electronic behavior, and has demonstrated the emergence of correlated insulating states and SC, offering new pathways for investigating quantum phenomena [16,17]. Doping, strain, and defect engineering further modulate conductivity, providing precise control over carrier concentration, mobility, and transport characteristics [48,49,50,51,52,53,54,55,56]. On other hand, the development of high-quality, large-sized 2D materials through chemical vapor deposition or novel exfoliation techniques [7,57,58,59,60,61,62] has enabled controlled studies of their transport properties, leading to enhanced device performance. Contact engineering, which improves interfaces between 2D materials and metal contacts, optimizes device performance by reducing contact resistance and enhancing charge injection [63,64,65,66,67,68,69,70,71,72].

Challenges remain, although advancements have been made in electrical transport studies and the novel electronic device development of 2D TMDs and vdW materials [73]. Synthesizing wafer-scale 2D materials with low defects remains a challenge for maintaining high transport properties. Achieving clean interfaces in 2D heterostructures is crucial for optimizing device performance. Controlled doping for uniform electrical response remains complex. Additionally, the oxidation of 2D materials in air presents a major issue in terms of reliability and consistency across research samples and devices. Exfoliating, stacking, and twisting 2D materials under ultra-high-vacuum (UHV) conditions may be necessary for model studies and high-quality device fabrication [74]. Theoretical models for predicting electrical transport often lack accuracy, hindering accurate predictions of device performance [75].

In this review, we focus on and draw attention to the intricate interplay among electrical transport, long-range ordered phases such as CDWs, SC, and magnetic order, and the role of disorders such as defects in these phases [76,77]. Transport, Charge Density Wave (CDW), SC, and magnetic order share the characteristic of being collective electronic behaviors, but differ in their phase coherence: while transport is phase-incoherent, CDWs, SC, and magnetic order exhibit long-range phase coherence. Despite their distinct macroscopic manifestations, these phases are fundamentally interconnected, arising from common microscopic underlying interactions such as electron–electron correlations and electron–phonon coupling [78,79,80]. Although these phases often coexist and interact within the same material, they are often studied separately. Investigating them together through a holistic and integrative perspective is essential, yet remains underexplored. Understanding the interdependence and mutual feedback between electrical transport, CDWs, SC, and magnetization, along with the role of defects is crucial for revealing the fundamental aspects of electron correlation and collective behavior, providing valuable insights for tuning and controlling correlated states in low-dimensional systems.

Electrical transport reflects the collective motion of charge carriers under external electric fields, governed by scattering, effective mass renormalization, and symmetry-dependent anisotropies arising from electron correlations and electron–phonon interactions [79,80,81]. Similarly, CDWs and SC arise from collective electron interactions, leading to spatially extended, phase-coherent structures, and are typically stabilized by electron–phonon interactions [79,82,83], whereas magnetism, where the spins of many electrons align in a coordinated manner, originates from spin–exchange interactions that can also be indirectly influenced by lattice vibrations and distortions [82,84,85].

Electrical transport properties are highly sensitive to the emergence of coherent electronic phases in 2D materials, serving as a key indicator of collective electron phase transitions to coherent states, such as CDWs, SC, or magnetism. These transitions trigger transport anomalies driven by carrier redistribution, symmetry breaking, altered scattering pathways, and electron pair condensation [76,79,86]. The electrical transport of 2D materials is fundamentally governed by their crystal structures and electronic band structures; particularly, the Fermi surface determines the carrier density of states (DOS), conductive channels, and carrier mobility [87,88,89,90,91,92,93]. The formation of CDWs, spin density waves (SDWs), and magnetically ordered states significantly impacts electrical transport properties in low-dimensional materials by inducing Fermi surface reconstruction, which affects both carrier density and mobility [94,95,96,97,98,99,100,101]. In TiSe_2_, a prototypical quasi-2D material, the CDW transition induces an abrupt Fermi surface reconstruction, accompanied by a minimum in both electron and hole mobilities. The resistivity of TiSe_2_ exhibits anomalous peaks that develop with the onset of the CDW phase, which broaden and shift to lower temperatures upon the intercalation of cobalt or iron atoms. This behavior is attributed to scattering from the softening CDW mode, highlighting the influence of CDW order on carrier dynamics and transport properties [96,102,103,104]. Similarly, in NdTe_3_, a quasi-1D material, the CDW phase generates new Fermi surface elements through band replicas, leading to quantum oscillations. This is associated with strong electron–phonon coupling, which affects the electronic structure and transport properties, including residual Fermi surface features within the CDW gap, impacting carrier dynamics [105]. In the over-doped cuprate Tl_2_Ba_2_CuO_6+x_, the CDW order emerges with a long correlation length, inducing a Fermi surface reconstruction that accompanies the transition into the CDW phase. This transition is associated with changes in carrier density and mobility, underscoring the role of CDW order in altering transport properties [106]. These examples illustrate that CDW formation in low-dimensional materials can significantly impact electrical transport by reconstructing the Fermi surface and altering carrier dynamics. In FeSe thin films, nematic fluctuations and spin–orbit coupling suppress carrier mobility and increase resistivity at low temperatures, highlighting the interplay between magnetic order and transport properties [107]. In CrI_3_ monolayers, the emergence of 2D ferromagnetism leads to large anisotropic magnetoresistance, attributed to spin-dependent scattering and symmetry breaking [108]. Similarly, in monolayer VSe_2_, an SDW-like transition results in a pronounced increase in resistivity due to the partial gapping of the Fermi surface and suppressed carrier conduction [109]. These examples demonstrate that magnetic and SDW order in 2D systems can substantially influence transport by modifying the electronic structure and altering carrier dynamics.

In turn, electrical transport not only serves as a sensitive probe for detecting the emergence of coherent electronic phases, but also as a tool to actively manipulate many-body correlated states in 2D materials. This control arises from the complex interplay between nonequilibrium carrier dynamics, modifications to the band structure, alterations in state occupations, electron–electron and electron–phonon interactions, Joule heating and energy dissipation, and phase transition mechanisms, all of which collectively influence the formation, stability, and dynamics of coherent electronic phases. When current flows through a material, it introduces a nonequilibrium charge distribution, which modifies the electron–phonon and electron–electron interactions that are central to many correlated states. Electrical current can directly influence the formation and stability of CDW phases in vdW 1T-TaS_2_. In-plane electrical current can induce transitions between different CDW phases, stabilizing one phase while suppressing another and disrupting the CDW order [110]. Additionally, transient current injection via localized voltage pulses can induce a metastable metallic phase from an insulating CDW ground state, directly triggering electronic and structural transitions. This phase transition results from current-driven carrier redistribution, which destabilizes the CDW phase through Joule heating and modifications to the material’s electronic structure [111]. These highlight the critical role of electrical current in controlling CDW dynamics and phase stability in 1T-TaS_2_. The role of current in redistributing electron occupation is also prominent in systems such as twisted bilayer graphene, where theoretical models suggest that current can control valley polarization and topological transport by altering the electronic band structure and filling of flat bands [112]. These examples illustrate the crucial role of electric current in tuning phase transitions, modifying electronic structure, and ultimately controlling coherent electronic phases in quantum materials. In the context of magnetism, electric currents can control magnetization through spin–transfer and spin–orbit torques, where spin-polarized charge carriers transfer angular momentum to localized magnetic moments, enabling magnetic switching without the need for external magnetic fields [113,114]. These findings demonstrate that conductive states in 2D materials facilitate spin–charge conversion, enabling the field-free electrical control of magnetization essential for spintronic applications [115,116]. Electrical transport properties, particularly carrier density and mobility, significantly control SC in 2D materials. In LaAlO_3_/SrTiO_3_ interfaces, electrostatic gating tunes carrier density and mobility, forming a SC dome and enabling phase transitions [117]. Similarly, in 1T-TaS_2_, gating modulates carrier density, affecting the balance between CDW and SC [118]. In monolayer NbSe_2_, ionic liquid gating adjusts carrier density, allowing the reversible control of SC and CDW order [119]. These examples highlight how electrical transport properties influence SC in 2D materials. In summary, electric transport actively manipulates coherent electronic phases by altering the charge carrier distribution, inducing nonequilibrium conditions and modifying the underlying interactions between electrons and phonons. These effects can lead to the stabilization or suppression of specific electronic phases, making electric transport an essential tool for controlling quantum states in next-generation electronic and quantum devices. Understanding these mechanisms is crucial for advancing material engineering and the design of devices that rely on the precise control of electronic phases.

Disorder in 2D materials critically affects their electronic and magnetic phases. In transport, defects scatter carriers and reduce mobility [120]; in CDWs, they pin or disrupt coherence [121]. Moderate disorder can enhance superconductivity by increasing DOS, though strong disorder suppresses it [122]. In magnetism, defects induce local moments or disturb magnetic order [123]. These effects are especially pronounced in low-dimensional systems, where sensitivity to disorder is heightened [77]. In graphene, point and topological defects such as divacancies and Stone–Wales transformations introduce localized π-states near the Fermi level, enhancing resonant scattering and reducing the mean free path. At sufficiently high defect densities, this disorder drives the system into an Anderson-localized regime characterized by a finite minimum conductivity [124]. Similarly, in semiconducting TMDs such as MoS_2_, intrinsic chalcogen vacancies generate deep-level states within the band gap, acting as carrier traps that degrade electrical transport by reducing mobility [77]. The spatial arrangement of defects in MoS_2_ also induces transport anisotropy by breaking in-plane symmetry, particularly when defects form aligned clusters or extended configurations [125]. Despite their detrimental effects on intrinsic transport properties, defects can be intentionally introduced and controlled—a strategy known as defect engineering—to tailor the electronic, magnetic, and optical responses of 2D materials for use in nanoelectronic and spintronic devices [126,127].

Here, in particular, we selectively highlight relevant results from our recent publications [128,129,130,131], with a refocused perspective on the interplay and correlation among electrical transport, collective electronic phenomena such as CDWs, magnetically ordered states, and local electronic defects in vdW materials, including ZrTe_3_ [128,129], 2H-TaS_2_ [131], and Cr_2_Si_2_Te_6_ [130]. These exhibit highly anisotropic electronic structures and are particularly sensitive to dopant-induced modifications at their layered interfaces. We demonstrate how foreign atom doping through intercalation, lattice substitution, and defect engineering within the 2D vdW interfaces modifies atomic and electronic interface structures, thereby influencing transport properties. Representative examples include quasi-one-dimensional lattice modifications in the 2D vdW interface of ZrTe_3_ via substitution and intercalation with Se, Hf, Cu, or Ni; the insertion of 2D magnetic atomic layers such as Mn, Fe, or Co into the vdW gap of 2H-TaS_2_; and short-range defect engineering at the vdW interface of Cr_2_Si_2_Te_6_. These methods provide effective strategies for tailoring electron transport by precisely engineering atomic and electronic environments at 2D vdW interfaces.

Beyond merely summarizing our published work, we present here a systematic, integrative, and comprehensive cross-analysis that offers complementary new insights, expanding, deepening, enhancing, supporting, and reinforcing the conclusions of the original works on electrical transport results. Specifically, we present a new point of view on how quasi-1D lattice modifications in ZrTe_3_ are associated with mixed valence states, variations in electron–phonon coupling strength, the onset temperature of CDWs, and transport behavior—ultimately leading to the proposal of an unconventional superconducting mechanism driven by mixed-valence-state fluctuations. These findings significantly advance the understanding of collective electronic behavior and SC in 2D systems. Additionally, we reveal that the evolution of out-of-plane magnetization in Cr_2_Si_2_Te_6_ significantly modulates in-plane charge transport, providing further evidence for spin–charge coupling and the magnetic control of electronic behavior in low-dimensional materials. These findings, derived from a comprehensive cross-analysis of related results, deepen our understanding of structure–property relationships in vdW materials and provide valuable insights for both fundamental research and the development of next-generation 2D nanoelectronics.

## 2. Electrical Transport Properties in Doped ZrTe_3_ [128,129]

The interplay between CDWs and SC is a central theme in the study of low-temperature electronic phenomena, reflecting fundamental aspects of electronic correlations and lattice instabilities. Both CDW and SC are manifestations of Fermi surface instabilities and low-temperature collective orders driven by strong electron–phonon interactions. These phenomena are particularly fascinating in layered vdW TMTCs, which are promising due to their ability to host quantum critical behaviors and their ease of exfoliation and integration into devices. Among these materials, ZrTe_3_ stands out as a compelling system for investigating the nuances and relationship between CDW and SC due to its unique electronic and structural properties.

**Figure 1:**
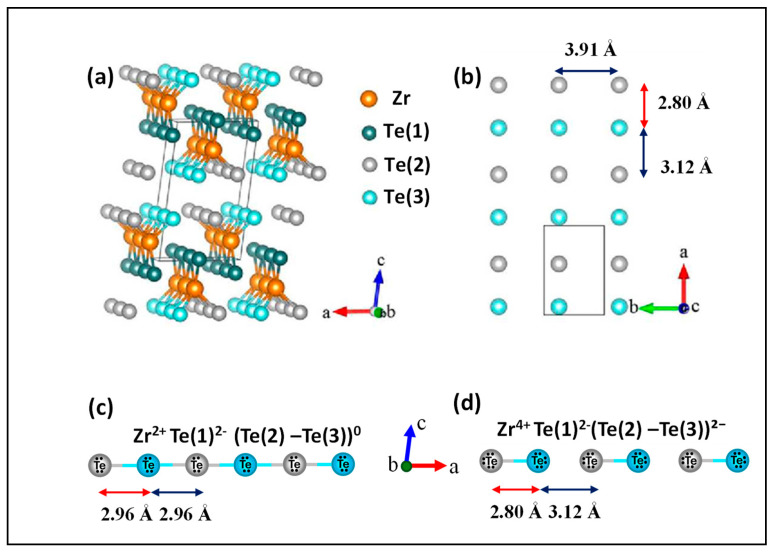
(**a**,**b**) Crystal structure of ZrTe_3_ with quasi-1D trigonal prismatic ZrTe_6_ chains along the b-axis and quasi-1D Te(2)-Te(3) atomic chains along the a-axis. (**c**) Valence state of Zr^2+^ Te(1)^2−^(Te(2)-Te(3))^0^, corresponding to Te(2)-Te(3) chains with equivalent intra- and inter-prism bonds and lattice distance of 2.96 Å [99]. (**d**) Valence state of Zr^4+^ Te(1)^2−^(Te(2)-Te(3))^2−^, corresponding to Te(2)-Te(3) chains without Te(2)-Te(3) inter-prism bonds due to a longer inter-prism Te(2)-Te(3) lattice distance compared to that of intra-prism Te(2)-Te(3) [99]. When the intra- and inter-prism Te(2)-T(3) distances lie between these extremes of (**c**,**d**), the valence state of the ZrTe_3_ atoms is expected to be somewhere between Zr^2+^ Te(1)^2−^(Te(2)-Te(3))^0^ and Zr^4+^ Te(1)^2−^(Te(2)-Te(3))^2−^ [99]. (**a**,**b**) are reprinted from [128] with the permission of AIP Publishing.

As illustrated in Figure 1a,b, the layered crystal structure of ZrTe_3_ consists of quasi-1D trigonal prismatic ZrTe_6_ chains with inversion symmetry propagating along the b-axis, as well as quasi-Te(2)-Te(3) atomic chains along the a-axis, making it a quasi-2D structure with a vdW gap in the a-b plane [47,132]. Band structure calculations [132,133] and angular-resolved photoemission spectroscopy (ARPES) measurements [134] reveal that the Fermi surface (FS) of ZrTe_3_ consists of a 3D hole-character FS sheet, primarily contributed by Zr d_y2_ orbitals, around the Brillouin zone center at the Γ point, and quasi-1D FS sheets aligned with the Brillouin zone boundary. ZrTe_3_ undergoes a nesting-type CDW transition below T_CDW_ = 63 K, opening a partial gap at the D electron pocket of the quasi-1D FS sheets with highly directional Te 5p_x_ orbitals from the Te(2)-Te(3) chain due to strong electron–phonon coupling between the D pocket electrons and the longitudinal vibration of the Te(2)-Te(3) lattices [135], while other parts of the FS remain unaffected. The Kohn anomaly, associated with a soft phonon mode and CDW fluctuations, has been identified [136].

Here, we demonstrate that the atomic, electronic, and vibrational structures of ZrTe_3_, along with its electrical transport properties, can be significantly modified through intercalation at the vdW gap and lattice substitution by foreign elements like Cu, Ni, Se, and Hf. By comprehensively reanalyzing these experimental results in conjunction with existing studies, we provide new insights that deepen our understanding of these observations, shedding light on the complex interplay between CDW and SC, as well as the mechanisms of SC in this intriguing material.

### 2.1. Dopant-Modulated Crystal Structures and Valence States

**Figure 2:**
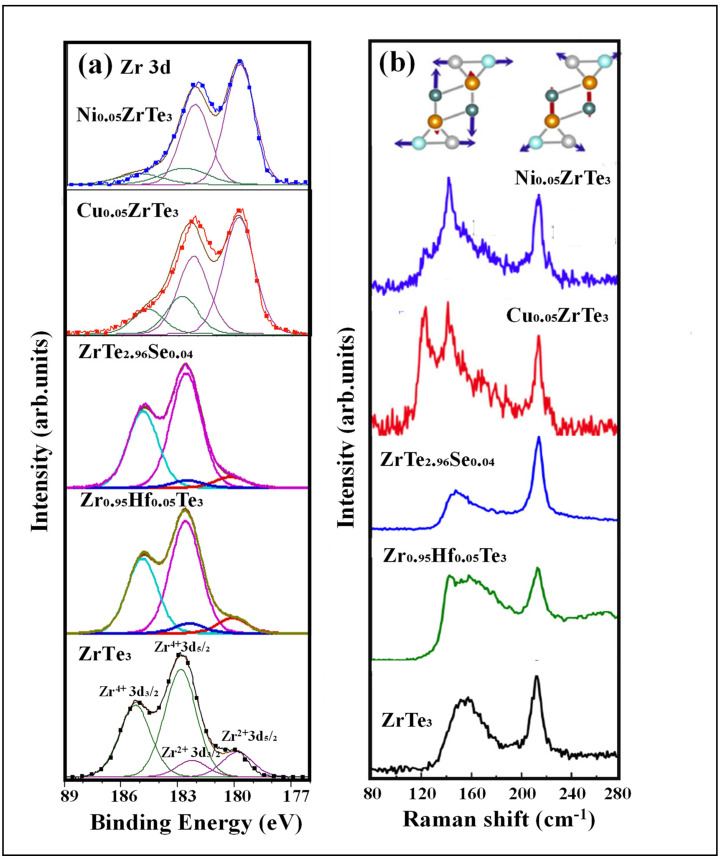
(**a**) Zr 3d XPS spectra obtained from ZrTe_3_, Zr_0.95_Hf_0.05_Te_3_, ZrTe_2.96_Se_0.04_, Cu_0.05_ZrTe_3_, and Ni_0.05_ZrTe_3_ crystals, respectively. (**b**) Corresponding Raman spectra of the indicated crystals. For pristine ZrTe_3_, vibrational patterns for the broad and sharp Raman peaks are shown above the peaks in the top panel, with arrows indicating the vibrational direction and lengths proportional to atomic displacements. All measurements were conducted at room temperature. ZrTe_3_, Cu_0.05_ZrTe_3_, and Ni_0.05_ZrTe_3_ in (**a**,**b**) are reprinted with permission from [127]. Copyright (2022) by the American Physical Society. Zr_0.95_Hf_0.05_Te_3_ and ZrTe_2.96_Se_0.04_ in (**a**,**b**) are reprinted from [128], with the permission of AIP Publishing.

As shown in Figure 2a, the Zr 3d XPS spectra reveal two distinct valence states for both pristine and doped ZrTe_3_, with a dopant-dependent mixed-valence-state ratio, as evidenced by two distinct sets of Zr 3d spin–orbit split doublets [129]. Based on the literatures, the spin–orbit split doublets at the lower binding energies of ~180 eV for Zr 3d_5_/_2_ and ~182.4 eV for Zr 3d_3_/_2_ correspond to Zr^2+^, while the doublets at higher binding energies of ~183 eV for Zr 3d_5_/_2_ and ~185.4 eV for Zr 3d_3_/_2_ are assigned to Zr^4+^ [137]. Theoretical analysis predicts that the valence state of ZrTe_3_ depends on the intra- and inter-prism Te(2)-Te(3) lattice distances, as shown in Figure 1c,d, representing two limiting cases [133]. An identical intra- and inter-prism Te(2)-Te(3) lattice distance results in equalized intra- and inter-prism Te(2)-Te(3) bonds, leading to a Zr^2+^ Te(1)^2−^[Te(2)-Te(3)]^0^ valence state (Figure 1c). In contrast, a longer inter-prism Te(2)-Te(3) distance compared to the intra-prism Te(2)-Te(3) distance results in the presence of only intra-prism Te(2)-Te(3) bonds, with the absence of inter-prism bonds, leading to a Zr^4+^ Te(1)^2−^[Te(2)-Te(3)]^2−^ valence state (Figure 1d). For pristine ZrTe_3_, the Zr^2+^ to Zr^4+^ ratio is approximately 25.1%, indicating the dominant presence of Zr^4+^. This corresponds to a regular intra-prism Te(2)-Te(3) dimer distance shorter than the inter-prism Te(2)-Te(3) distance, consistent with the theoretically predicted lowest energy structural model, as shown in Figure 1a,b.

XPS analysis of Zr_0.85_Hf_0.15_Te_3_ and ZrTe_2.96_Se_0.04_ synthesized by substituting Zr with Hf or Te with Se shows decreased Zr^2+^/Zr^4+^ ratios of 8.8% and 13.5%, respectively, indicating a reduced [Te(2)-Te(3)]^0^ to [Te(2)-Te(3)]^2−^ ratio. This suggests less stretched intra-prism Te(2)-Te(3) lattices, which is consistent with X-ray absorption near edge spectroscopy (XANES) data showing a decrease in the average unit cell size for ZrTe_2.96_Se_0.04_ and Zr_0.85_Hf_0.15_Te_3_ compared to undoped ZrTe_3_ [88]. The electron affinity order is Se > Te > Zr > Hf, with selenium being more electron-affine than tellurium, and hafnium more prone to electron loss than zirconium. This trend explains the lower Zr^2+^/Zr^4+^ ratio observed when substituting tellurium with selenium or zirconium with hafnium. In contrast, for Cu_0.05_ZrTe_3_ and Ni_0.05_ZrTe_3_, synthesized by introducing 5% Cu or Ni into the vdW gap, the XPS spectra show an increased Zr^2+^ to Zr^4+^ ratio compared to pristine ZrTe_3_, rising to 68.0% and 77.0%, respectively [128]. This increase suggests an enhanced valence transition from [Te(2)-Te(3)]^2−^ to [Te(2)-Te(3)]^0^, resulting from the expansion of intra-prism Te(2)-Te(3) distances in the quasi-1D chains. These findings align with the XANES results, which show an expansion of the a- and c-axis lattice parameters by approximately 0.2–0.3% for Cu_0.05_ZrTe_3_ and 0.6–0.7% for Ni_0.05_ZrTe_3_, respectively, while the b-axis remains relatively unchanged [128]. The Te(2)-Te(3) lattice distortions, reduced by foreign atom interactions and substitutions as suggested by XPS, are further confirmed by Raman spectroscopy, as described below.

### 2.2. Dopant-Modulated Vibrational Structures

As shown in Figure 2b, for pristine ZrTe_3_, the Raman spectra show prominent peaks around 215 cm^−1^ and 155 cm^−1^. The broad peak at 155 cm^−1^ corresponds to the longitudinal vibration of the Te(2)-Te(3) lattice along the a-axis direction, and this vibration is strongly coupled with quasi-1D Fermi electrons at the D pocket, primarily involving Te 5p_x_ orbitals, leading to the broadening of the peak [135]. In contrast, the sharp peak at 215 cm^−1^ indicates good crystal quality and less phonon coupling with Fermi electrons [135].

Upon substituting Zr with Hf, the 155 cm^−1^ peak broadens, and its intensity ratio to the sharp 128 cm^−1^ peak increases compared to pristine ZrTe_3_, suggesting enhanced quasi-1D Fermi electron–phonon coupling. This behavior is attributed to the more available Raman-active phonons involving regular intra-prism Te(2)-Te(3) dimers, rather than the expanded intra-prism Te(2)-Te(3) lattice, as indicated by the higher ratio of [Te(2)-Te(3)]^2−^ to [Te(2)-Te(3)]^0^ observed in XPS.

For ZrTe_2.96_Se_0.04_, where Se partially substitutes Te in the Te(2)-Te(3) chains, the broader Raman peak with an asymmetric Fano lineshape at 155 cm^−1^ indicates strong electron–phonon coupling, similar to pristine ZrTe_3_ and Zr_0.95_Hf_0.05_Te_3_. However, the intensity ratio of the broad 155 cm^−1^ peak to the sharp 128 cm^−1^ peak decreases substantially, suggesting a reduced number of available Raman-active phonons for electron–phonon coupling. Similar to Zr_0.95_Hf_0.05_Te_3_, the Raman-inactive phonons caused by the stretched intra-prism Te(2)-Te(3) lattice decrease, as indicated by the increased ratio of [Te(2)-Te(3)]^2−^ to [Te(2)-Te(3)]^0^ in the XPS results. The overall reduction in the intensity of the broad 155 cm^−1^ peak is likely due to the Se-substituted Te(2)-Te(3) chains being Raman-inactive, as Se’s lower atomic mass alters the phonon modes and induces chain disorder and distortion.

In contrast, upon the intercalation of Cu and Ni at the vdW gap, the Raman spectra of Cu_0.05_ZrTe_3_ and Ni_0.05_ZrTe_3_ show new, sharp, red-shifted peaks at 143 cm^−1^ and 125 cm^−1^, indicating intra-prism Te(2)-Te(3) lattice expansion, consistent with the XPS and XANES analyses. The sharpness of these red-shifted peaks suggests less phonon coupling with quasi-1D electrons due to lattice expansion. With a decreased ratio of [Te(2)-Te(3)]^2−^ to [Te(2)-Te(3)]^0^, as suggested by XPS, fewer unstretched intra-prism Te(2)-Te(3)] dimers are available for electron coupling, significantly reducing the overall intensity at 155 cm^−1^.

The XPS and Raman results indicate that the intercalation of foreign atoms into the 2D vdW gap in the a-b plane, or the substitution of Te or Zr atoms, alters the intra- and inter-prism Te(2)-Te(3) distances, significantly affecting the coupling between the quasi-1D Fermi electrons and the longitudinal vibrations of the Te(2)-Te(3) phonons. Consequently, this influences the CDW transition temperature T_CDW_ and the degree of CDW formation, which, in turn, impacts the temperature-dependent electrical transport behavior, as discussed below.

### 2.3. Interplay Among Electron–Phonon Coupling, CDW, and Electrical Transport

**Figure 3:**
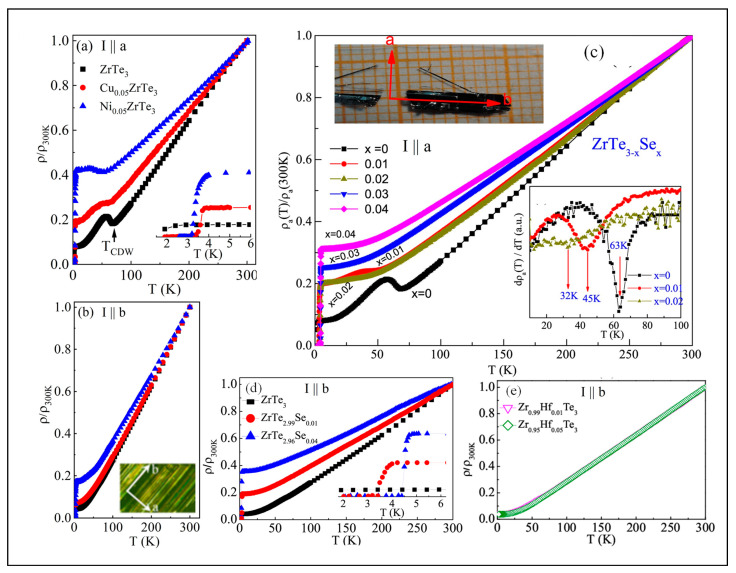
The temperature dependence of normalized resistivity, ρ(T)/ρ(300 K), with current applied along the a-axis and b-axis, as marked by I ‖ a and I ‖ b, respectively. Panels (**a**,**b**) show data for ZrTe_3_, Cu_0.05_ZrTe_3_, and Ni_0.05_ZrTe_3_. The inset in (**a**) highlights the low-temperature superconducting transitions. Panels (**c**,**d**) show data for ZrTe_3−x_Se_x_ with varying doping levels (x). The upper inset in (**c**) shows a photograph of cleaved ZrTe_3−x_Se_x_ crystals, with fibers visible along the b-axis. The lower inset in (**c**) displays the CDW transition temperature, T_CDW_, marked by an arrow, determined from the dips in the differential ρ/ρ(300K) − T curves. The inset in (**d**) shows the low-temperature ρ/ρ(300K) − T behavior, indicating the superconducting transition temperature Tsc. Panel (**e**) shows data for Zr_1−x_Hf_x_Te_3_. (**a**,**b**,**d**) are reprinted with permission from [127]. Copyright (2022) by the American Physical Society. (**c**) is adapted from [137], licensed under CC BY 4.0. (**d**,**e**) are reprinted from [128], with the permission of AIP Publishing.

Figure 3 illustrates the temperature dependence of normalized resistivity, ρ(T)/ρ(300K), for ZrTe_3_, Cu_0.05_ZrTe_3_, Ni_0.05_ZrTe_3_, Zr_0.95_Hf_0.05_Te_3_, and ZrTe_2.96_Se_0.04_, with current applied along the a-axis (a) and b-axis (b), respectively [128,129,138]. Pristine ZrTe_3_ exhibits metallic behavior, with resistivity decreasing as temperature decreases. An upturn in resistivity is observed at the CDW formation temperature of 63 K along the a-axis, marked by a minimum in dρ/dT, while no upturn is seen along the b-axis. The parallel quasi-1D band associated with the quasi-1D Te(2)-Te(3) atomic chains and the 3D-like peanut-shaped hole pocket around the Γ-point on the Fermi surface [134] are responsible for the metallic behavior. Strong electron–phonon coupling between the quasi-1D Fermi electrons at the D pocket and the quasi-1D Te(2)-Te(3) longitudinal vibrations, as indicated by Raman spectroscopy, partially opens an energy gap in the quasi-1D band at the D pocket, accompanied by CDW formation [135], leading to the anisotropic resistivity upturn along the a-axis. Following the CDW transition, SC emerges with an onset at T_SC_≈2K, as shown in Figure 3a.

Compared to pristine ZrTe_3_, CDW formation is suppressed, accompanied by enhanced SC in Cu_0.05_ZrTe_3_ and Ni_0.05_ZrTe_3_ as shown in Figure 3a. This is indicated by a reduced resistivity upturn, lower CDW transition temperatures T_CDW_ ≈ 58 K for Cu_0.05_ZrTe_3_ and 41 K for Ni_0.05_ZrTe_3_, and increased superconducting onset temperatures T_SC_ ≈ 3.6 K for Cu_0.05_ZrTe_3_ and 3.1 K for Ni_0.05_ZrTe_3_, respectively. The residual resistivity ratio (RRR) (RRR = ρ_300K_/ρ_5k_) decreases from 12.7 in ZrTe_3_ to 5.3 in Cu_0.05_ZrTe_3_ and 2.4 in Ni_0.05_ZrTe_3_, indicating increased disorder scattering from Cu or Ni intercalation at the 2D vdW gap. The suppressed CDW is due to weakened coupling between the quasi-1D electron at the D pocket and the Te(2)-Te(3) longitudinal phonons, caused by the increased intra-prism lattice distance of Te(2)-Te(3) from Cu or Ni intercalation, as indicated by the Raman and XPS results.

The temperature-dependent resistance of ZrTe_2.99_Se_0.04_, as shown in Figure 3c, reveals no CDW-induced resistivity anomaly hump and exhibits an increased zero-resistivity onset temperature of 4.4 K, as shown in Figure 3d, indicating the complete suppression of long-range CDW phase coherence and enhanced SC [138]. The suppression of both CDW order and the resistance anomaly results in phonons in the Se-substituted Te(2)-Te(3) chains failing to couple with the quasi-1D Fermi electrons, along with chain disorder caused by Se’s lower atomic mass compared to Te. In contrast, Zr_0.95_Hf_0.05_Te_3_ exhibits an increased CDW transition temperature of 72 K [138], with no observable SC above 2 K, as shown in Figure 3e, suggesting that hafnium substitution for zirconium stabilizes CDW while inhibiting SC. Substituting Zr with Hf increases the Raman-active unstretched intra-prism Te(2)-Te(3) dimers, as suggested by XPS. This enhances the electron–phonon coupling between the quasi-1D electron at the D pocket and the Te(2)-Te(3) longitudinal vibrational mode, as indicated by the Raman results, consequently stabilizing CDW formation and suppressing SC.

Foreign atom intercalation or lattice atom substitution in the 2D vdW gap alters the intra-and inter-prism Te(2)-Te(3) distance, impacting the coupling between the quasi-1D Fermi band and the longitudinal vibrations of the Te(2)-Te(3) chains, thereby affecting CDW formation and the onset temperatures for CDW and SC. Additionally, a competitive relationship exists between CDW and SC, where CDW suppression typically enhances SC, and vice versa. In the following, we will discuss how the longitudinal vibration mode of Te(2)-Te(3) chains impacts SC.

### 2.4. New Insights into the Superconductivity Mechanism of ZrTe_3_

Upon CDW formation, 1D Fermi electrons accumulate at the van Hove singularity (vHS) near the B-pocket of the Brillouin zone, at the expense of a gap opening at the D-pocket, as observed by ARPES [134]. The high density of states at the vHS saddle point on the Fermi surface strengthens electron correlations and drives Fermi surface instability, promoting bosonic and/or Cooper pair condensation. In addition, the vHS simultaneously hosts both narrow quasi-1D Te 5*p*_x_ and wide 3D-like Zr *dz* orbitals [133]. ZrTe_3_ has been experimentally demonstrated to exhibit ‘mixed filament–bulk superconductivity’, and the vHS is proposed to play a key role in this phenomenon [139]. At the vHS, local pairs with Bose-like characteristics and short coherence lengths, initially formed on the narrow quasi-1D Fermi band, are transferred to the wider 3D Fermi band, where they form conventional Cooper pairs with longer coherence lengths, leading to bulk SC. This represents a crossover between filamentary and bulk SC as temperature varies.

Our XPS results suggest that doping induces spatially mixed valence states of Zr^4+^Te(1)^2−^[(Te(2)-Te(3))]^2−^ and Zr^2+^Te(1)^2−^[Te(2)-Te(3)]^0^. In fact, for pristine, doping-free ZrTe3, DFT calculations indicate that compressing or stretching the intra-prism Te(2)–Te(3) distances along the *a*-axis, with each Te atom displaced by a maximum of 0.08 Å from its equilibrium position, induces dynamic valence fluctuations between Zr^4+^Te(1)^2−^(Te(2)–Te(3))^2−^ (2.80 Å intra-prism and 3.12 Å inter-prism lattice distances) and Zr^2+^Te(1)^2−^(Te(2)–Te(3))^0^ (equalized intra- and inter-prism distances of 2.96 Å), accompanied by the redistribution of Fermi electron pairs between the quasi-1D electron bands and the 3D hole pocket around Γ [133]. This provides new microscopic insight into the crossover between filamentary and bulk SC—specifically, how the transfer of quasi-1D electron pairs to the 3D band is triggered at the vHS.

We propose here that the ‘mixed filament–bulk superconductivity’ observed in ZrTe_3_ is associated with ‘mixed-valence-state fluctuations’ between Zr^4+^Te(1)^2−^(Te(2)-Te(3))^2−^ and Zr^2+^Te(1)^2−^(Te(2)-Te(3))^0^. These fluctuations lead to dynamic local electron pair transfer between the Te(2)-Te(3) chains and Zr atoms via a hybrid quasi-1D + 3D vHS, corresponding to a fluctuating redistribution of electrons between the narrow quasi-1D band and the wide 3D-like band at the vHS. These fluctuations require only a 0.08 Å displacement of each Te(2)/Te(3) atom in the breathing phonon mode, suggesting a low reorganization energy for both lattice and electronic rearrangement. Filamentary SC, characterized by a short coherence length, corresponds to local electron pair hopping along the a-axis, which is equivalent to the propagation of valence state switching, with a charge difference of two, between Zr^4+^Te(1)^2−^(Te(2)-Te(3))^2−^ and Zr^2+^Te(1)^2−^(Te(2)-Te(3))^0^ along the a-axis under an external electrical field.

A recent discovery revealed that mixed valence states and potential valence fluctuations in metal ions may play a crucial role in the manifestation of CDW order and SC in perovskite antimonates such as Ba_1−x_K_x_SbO_3_ [140]. The compound exhibits a CDW order at x = 0−0.5 with spatially alternating Sb^3+^ and Sb^5+^ valence states and emerges into superconducting upon CDW suppression at x = 0.65, with all Sb sites reaching an equivalent intermediate valence of +4.65.

According to the Mixed Valence Model for SC [141,142], which involves three consecutive valence states, electron pairing is directly related to the existence of two valence states being different in two charges. The electron pair delocalizes from the localized state via an intermediate transition valence state, rather than directly exchanging electron pairs between two valence states that are different in two charges. This is because direct exchange demands more reorganization energy for lattice and electronic structure rearrangement compared to indirect exchange via an intermediate valence state.

During longitudinal vibrations of the Te(2)–Te(3) chains at intermediate distances between the two extreme valence states—Zr^4+^Te(1)^2−^(Te(2)–Te(3))^2−^ (no inter-prism bond) and Zr^2+^Te(1)^2−^(Te(2)–Te(3))^0^ (equal inter- and intra-prism bonds), as shown in Figure 1c—an intermediate valence state, Zr^3+^Te(1)^2−^(Te(2)–Te(3))^1−^, can be expected to emerge when the inter-prism bond is present but weaker than the intra-prism bond. This intermediate state of Zr^3+^Te(1)^2−^(Te(2)-Te(3))^1−^ facilitates electron delocalization through a disproportionation reaction:

- In the bulk: 2Zr^3+^ ↔ Zr^2+^ + Zr^4+^;
- In the quasi-1D lattice: 2(Te(2)-Te(3))^1−^ ↔ (Te(2)-Te(3))^2−^ + (Te(2)-Te(3))^0^.

It was proposed that SC can emerge when the Mott–Hubbard parameter U, which is the free energy of the disproportionation reaction, is close to zero and the coupling energy between the two valence states different by two charges exceeds the reorganization energy required for the lattice and electronic structure rearrangement [141,142,143,144]. The condition for SC emergence is satisfied in the disproportionation reaction of ZrTe_3_, as follows: First, each Te(2)/Te(3) deformation associated with electron delocalization via the intermediate valence state is less than 0.08 Å, that is, the maximum displacement occurring during direct electron pair delocalization between Zr^4+^Te(1)^2−^(Te(2)-Te(3))^2−^ and Zr^2+^Te(1)^2−^(Te(2)-Te(3))^0^ valence states. This results in lower reorganization energy for lattice and electronic rearrangement during the disproportionation reaction. Second, the disproportionation reaction and coupling between the two valence states, Zr^4+^Te(1)^2−^[(Te(2)–Te(3))]^2−^ and Zr^2+^Te(1)^2−^[Te(2)–Te(3)]^0^, occur spontaneously during thermal or even zero-point vibrations of Te(2)–Te(3) due to small lattice displacements, indicating the small free energy of the reaction and strong coupling between the two valence states different by two charges. In addition, the disproportionation reaction implies that electron pair transfer between the quasi-1D and 3D-like Fermi bands is not restricted in an intra-prism. It can instead be transferred between two prisms separated by a long distance, with one electron transferred in each prism via the intermediate valence state of Zr^3+^Te(1)^2−^(Te(2)-Te(3))^1−^. The coherent delocalization of electrons from widely separated sites gives rise to the bulk SC state with long coherence length in ZrTe_3_. This process of bulk SC emergence is facilitated by valence fluctuations [121,145,146,147,148] among three consecutive valence states at the vHS between the quasi-1D and 3D Fermi bands, driven by longitudinal vibrations of the Te(2)–Te(3) lattice. The high DOS at the vHS enhances electron correlation, promoting the superposition of electron wavefunctions from these three fluctuating valence states. This superposition leads to wavefunction overlap and interference, establishing the long-range quantum coherence of electron pairs. The resulting final delocalized vibronic wavefunction forms a gapped ground state, allowing condensed Cooper pairs to move without resistance [141,142]. SC emerges as the system lowers its energy by forming Cooper pairs rather than allowing transitioning between valence states, rendering valence-state differences unobservable [141,149,150,151].

Filamentary-to-bulk SC transition aligns with the concept of the Bose–Einstein Condensate (BEC) to Bardeen–Cooper–Schrieffer (BCS) crossover, where the system transitions from a Bose-like state of tightly bound pairs to a BCS-like state of loosely bound Cooper pairs. Here, we propose that the emergence of bulk SC is driven by valence-state instability at the vHS [151], as the consequence of soft Te(2)–Te(3) lattice longitudinal vibrations at a critical point where the superposition of these valence fluctuations becomes pronounced.

Unlike the BCS theory of conventional SC, where electron–phonon coupling is crucial for Cooper pair formation and stabilization, electron pairing in ZrTe_3_ is directly related to two valence states differing by two charges, with Cooper pairs forming through the vHs, rather than being phonon-stabilized, making the pairing mechanism unconventional. However, phonons associated with the Te(2)-Te(3) longitudinal vibration still play a crucial role in this unconventional SC. First, phonons from the longitudinal vibration of Te(2)-Te(3), coupling with 1D Fermi electrons at the D-pocket, lead to electron accumulation at the vHs, initiating the condensation of quasi-1D electron pairs into bosons/Cooper pairs. Second, phonon-driven valence-state fluctuations trigger electron transfer between quasi-1D and 3D Fermi bands at the VHS, which is responsible for the crossover between filamentary and bulk SC. Third, coherent electron pair delocalization from two spatially wider, separated prisms is facilitated through a disproportionation reaction associated with phonon-induced valence fluctuations among three consecutive valence states. Here, phonon-induced mixed-valence-state fluctuations in ZrTe_3_ shed light on unconventional SC mechanisms and may help advance high-temperature SC.

The electrical transport behavior of layered ZrTe_3_ exhibits a pronounced dependence on thickness, driven by the interplay among CDW order, SC, and dimensional effects. In bulk form, ZrTe_3_ shows a CDW transition near 63 K and filamentary SC below 2 K. When the thickness of ZrTe_3_ is reduced to around 18 nm, the CDW transition is suppressed and SC is enhanced, with the transition temperature increasing to nearly 5 K [152]. However, as the thickness is further reduced below approximately 8 nanometers, the CDW transition reappears at a higher temperature, while SC vanishes. The observed behaviors can be attributed to the shifting of the Fermi level with decreasing thickness, the anisotropic strain induced by the substrate, and enhanced disorder scattering as the system approaches the 2D limit. In addition, ZrTe_3−x_ nanoplates with controlled levels of disorder, such as tellurium vacancies, show enhanced SC and reduced CDW order [153]. In these disordered thin films, a 2D superconducting state emerges with characteristics consistent with a Berezinskii–Kosterlitz–Thouless transition. These findings highlight the delicate balance between competing electronic orders in low-dimensional systems and underscore the potential for tuning material properties through thickness modulation and controlled disorder.

## 3. Electrical Transport in vdW Magnets: 2H-M_x_TaS2 (M = Mn, Co) [131]

**Figure 4:**
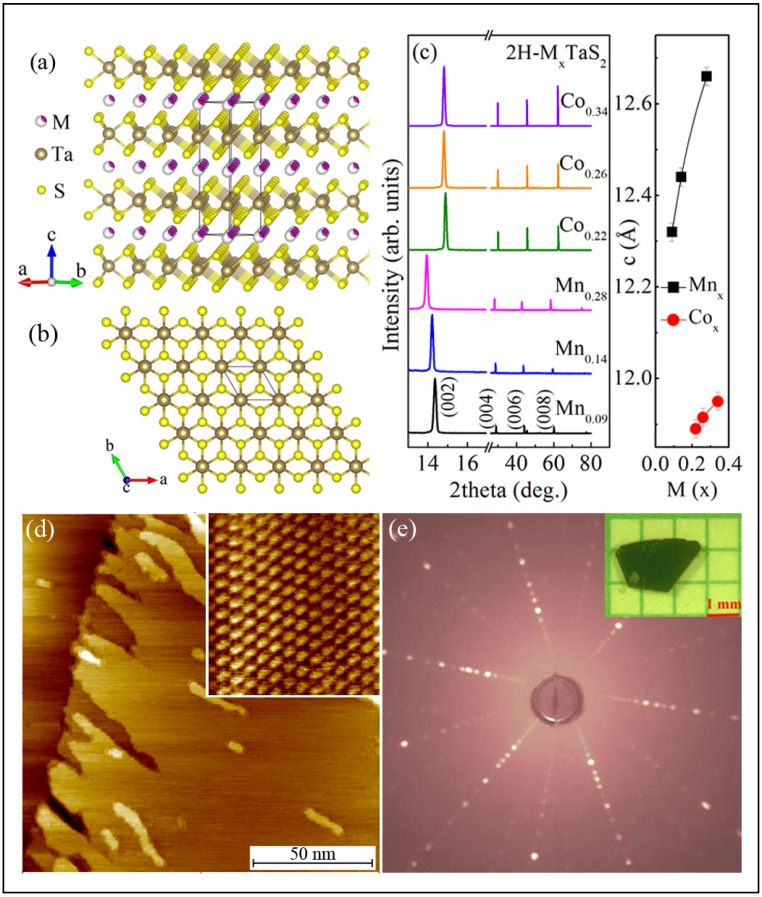
Crystal structure of 2H-M_x_TaS_2_ (M = Mn, Co) viewed from (**a**) the side and (**b**) the top. (**c**) Single-crystal XRD patterns of 2H-M_x_TaS_2_ (M = Mn, Co) and the corresponding evolution of the lattice parameter *c* with varying intercalation content *x*. (**d**) STM topography of the surface of 2H-Mn_0.28_TaS_2_. (**e**) Laue X-ray pattern of the 2H-Co_0.34_TaS_2_ crystal surface, highlighting the sixfold symmetry of the hexagonal structure. (**a**–**e**) are reprinted from [131], licensed under CC BY 4.0.

The recent discovery of intrinsic long-range magnetic order in ultrathin 2D van vdW magnets, such as FePS_3_, Cr_2_Ge_2_Te_6_, CrI_3_, Fe_3_GeTe_2_, VSe_2_, MnSe_2_, and Fe_3+x_GaTe_2_, has spurred extensive research aimed at understanding the underlying physical mechanisms and optimizing the functionalities of vdW heterostructures and devices [47,154,155,156,157,158,159,160,161]. Intercalated transition metal dichalcogenides, which typically feature 3d metal atoms within the vdW gap, exhibit a wide range of magnetic behaviors [162,163]. A detailed study of the electrical transport properties of 2H-M_x_TaS_2_ (M = Mn, Co) single crystals, with M atoms intercalated into the 2D vdW gap, during magnetic-order evolution is essential for understanding the interplay between spin correlations and electrical transport, as well as for advancing spintronic applications. Figure 4a,b present the crystal structure of 2H-M_x_TaS_2_ (M = Mn, Co) with space group P6322 from side and top views, respectively [131]. X-ray diffraction (XRD) 2θ scans (Figure 4c) show sharp peaks indexed to (00l) planes, indicating that the plate surfaces of the single crystals are oriented perpendicular to the c-axis. As the x-value increases, the (00l) peaks shift to lower angles, which is particularly noticeable in the enlarged (002) peak, suggesting an expansion of the lattice parameter c. The values of c, obtained from Bragg’s law, increase monotonically with x, confirming the intercalation of M atoms and expansion of the vdW gap in 2H-TaS_2_. Figure 4d displays the scanning tunneling microscopy (STM) topography of a representative 2H-Mn_0.28_TaS_2_ crystal surface, revealing a triangular lattice. The Laue XRD pattern of a 2H-Co_0.34_TaS_2_ crystal (Figure 4e) confirms the hexagonal symmetry and well-defined orientation along the (00l) direction. The intercalation of M atoms induces distinct magnetic susceptibility χ(T) behavior in 2H-M_x_TaS_2_ under an applied magnetic field parallel to the ab-plane or the c-axis at H = 1 kOe, in both zero-field (ZFC) and field-cooling (FC) modes [131]. In 2H-Mn_0.28_TaS_2_, ferromagnetism (FM) with easy-plane anisotropy develops, with a paramagnetic (PM)-to-FM transition at a temperature T_C_ of approximately 82 K.

**Figure 5:**
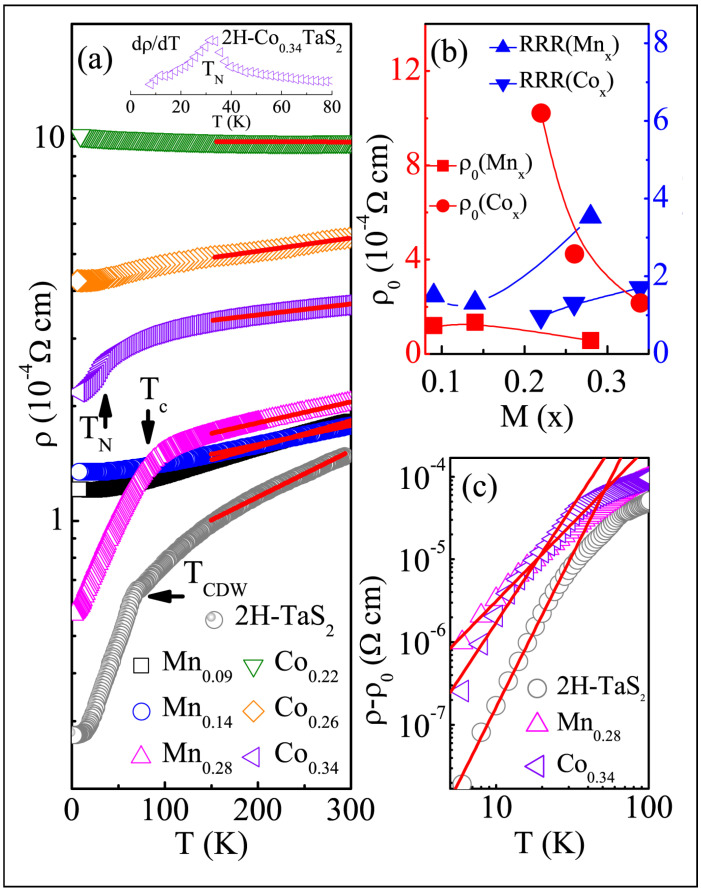
(**a**) Temperature dependence of in-plane resistivity ρ(T) for 2H-M_x_TaS_2_ (M = Mn, Co) single crystals. The inset shows dρ/dT for 2H-Co_0.34_TaS_2_ around T_N_. (**b**) Evolution of residual resistivity ρ_0_ (left axis) at T = 4 K and RRR = ρ_300_K/ρ_4_K (right axis) for 2H-M_x_TaS_2_ (M = Mn, Co). (**c**) Temperature-dependent ρ − ρ_0_ for 2H-TaS_2_, 2H-Mn_0.28_TaS_2_, and 2H-Co_0.34_TaS_2_, with power-law fits from 30 to 4 K (solid lines). (**a**–**c**) are reprinted from [131], licensed under CC BY 4.0.

In contrast, 2H-Co_0.22_TaS_2_ exhibits FM with strong uniaxial anisotropy along the c-axis, which evolves into three-dimensional antiferromagnetism (AFM) in 2H-Co_0.34_TaS_2_, with a Néel temperature T_N_ of around 36 K. Figure 5a illustrates the temperature dependence of in-plane resistivity, ρ(T), for 2H-MxTaS_2_ (M = Mn, Co) single crystals [131]. The intercalation of M atoms suppresses the CDW transition at 78 K observed in 2H-TaS_2_ [164]. The residual resistivity, ρ_0_, at 4 K and the residual resistivity ratio (RRR = ρ_300K_/ρ_4K_) are 2.76 × 10^−5^ Ω·cm and 5.4 for 2H-TaS_2_, respectively.

All M-intercalated samples exhibit metallic behavior, with higher ρ_0_ values and smaller RRRs compared to 2H-TaS_2_. The evolution of ρ_0_ and RRR as a function of intercalation content is shown in Figure 5b. Initially, ρ_0_ is high, likely due to incomplete ordering of the Mn or Co atoms. However, as the Mn or Co content increases, ρ_0_ decreases and RRR increases. The most ordered 2H-Mn_0.28_TaS_2_ and 2H-Co_0.34_TaS_2_ crystals may form a Mn or Co superstructure within the vdW gap, which could be confirmed by electron diffraction measurements. This ordering likely reduces electrical resistivity. All samples exhibit a nearly linear temperature dependence of resistivity in the high-temperature paramagnetic regime (Figure 5a). The resistivity slope shows a sharp change in magnitude at T_C_ for 2H-Mn_0.28_TaS_2_, while a distinct kink is observed around T_N_ for 2H-Co_0.34_TaS_2_, as shown in the derivative plot dρ/dT in the inset of Figure 5a [131]. This suggests significant coupling between the transport carriers in the TaS_2_ planes and the local moments of the intercalated M atoms, especially in higher-M-content samples. Below the magnetic transition temperatures, ρ(T) decreases more steeply with decreasing temperature for 2H-Mn_0.28_TaS_2_ and 2H-Co_0.34_TaS_2_ due to reduced spin-disorder scattering [165]. After subtracting the residual resistivity, the low-temperature resistivity (ρ − ρ_0_) for 2H-TaS_2_, 2H-Mn_0.28_TaS_2_, and 2H-Co_0.34_TaS_2_ is plotted in Figure 3c. A power-law fit (∝Tᵅ) below 30 K yields α = 3.7 for 2H-TaS_2_, 1.9 for 2H-Mn_0.28_TaS_2_, and 2.8 for 2H-Co_0.34_TaS_2_, respectively. In 2H-TaS_2_, the low-temperature resistivity is dominated by electron–phonon (T^5^) scattering and contributions from electron scattering by collective CDW excitations (T^2^). For 2H-Mn_0.28_TaS_2_ and 2H-Co_0.34_TaS_2_, the resistivity follows a quasi-quadratic temperature dependence, likely due to spin–wave scattering. Above T_C_ and T_N_, where the spins are disordered, the magnetic scattering is expected to be temperature-independent; the linear T-dependence of resistance is therefore dominated by electron–phonon scattering.

The above illustrates that when 2D magnetic atomic layers are inserted into the vdW gaps, the gap can be intentionally expanded by increasing intercalant concentration, effectively tuning the interlayer coupling between intrinsically coupled TaS_2_ monolayers and enhancing their 2D characteristics. A strong interdependence is demonstrated between CDW order, magnetic order, and electrical transport. Intercalated magnetic atoms induce spin-dependent electron scattering, quenching CDW coherence and modifying transport properties. Electrical transport evolves accordingly as disordered local moments transition into long-range magnetic order. Thus, electrical transport serves as a sensitive probe of both the emergence of magnetic correlations and their impact on CDW ordering, underscoring the intricate interplay between spin, charge, and their collective dynamics.

The disordered intercalation of Co atoms in the van der Waals gaps of 2H-Co_0.27_TaS_2_ show a clear semiconducting-like behavior [166]. This is governed by a variable-range hopping (VRH) mechanism. In this regime, electrons are no longer delocalized, but instead become trapped in spatially and energetically random localized states created by Co intercalation. As thermal energy assists electrons in hopping between these localized sites, the overall conductivity becomes increasingly sensitive to temperature and disorder, making hopping the dominant transport mechanism. The system’s electrical behavior is thus determined less by intrinsic band structure and more by disorder-induced localization effects.

A study on ultrathin 2H-TaS_2_ revealed that as the thickness of the material is reduced to the few-layer limit (~3.5 nm), its SC transition temperature increases significantly from about 0.5 K in the bulk to approximately 2.2 K [167]. This unexpected enhancement of SC, in contrast to the typical suppression in reduced dimensions, was attributed to stronger electron–phonon coupling in thinner samples, as supported by theoretical calculations. As 2H-TaS_2_ is thinned down to the monolayer limit, SC is significantly enhanced, with the SC transition temperature Tc increasing to 3.4 K in monolayers [168]. This enhancement coincides with the suppression of the CDW phase, whose transport signatures diminish and eventually vanish as the material approaches the 2D limit. Electronic structure calculations show that the weakening of the CDW order leads to an increased DOS at the Fermi level, which in turn promotes stronger SC. These results underscore a clear competition between CDW order and SC in 2H-TaS_2_, where reduced thickness suppresses CDW coherence and facilitates the emergence of a more robust SC state. The findings highlight the critical influence of dimensionality on quantum electronic phases and demonstrate a promising strategy for engineering superconductivity in 2D layered materials and understanding correlated electronic phases in reduced dimensions.

## 4. Short-Range Crystalline Order-Tuned Conductivity in Cr_2_Si_2_Te_6_ vdW Magnetic Crystals [130]

Defects, dislocations, and other crystalline imperfections in 2D materials beyond graphene often introduce interesting physics and useful functionality. In particular, crystalline imperfections in 2D magnetic materials can significantly influence their conducting and magnetic properties. Of particular interest are the conducting electronic states in 2D magnetic materials that can be used for spin–charge conversion in spintronic devices [113,115,116,130,169]. The FM state in Cr_2_X_2_Te_6_ (X = Si, Ge) persists even in monolayer and few-layer crystals, as demonstrated both theoretically and experimentally [170,171]. Surface Ge vacancies induce a metallic state by closing the band gap in Cr_2_Ge_2_Te_6_ multilayers, resulting in an increase in magnetic anisotropy energy [172]. Cr_2_Si_2_Te_6_ exhibits magnetic order in few-layer crystals. Defects in Cr_2_Si_2_Te_6_ are predicted to result in a fully spin-polarized current arising from a bipolar magnetic semiconducting state [173]. However, its large band gap limits electronic conduction. Here, we show that defect-induced short-range crystal ordering in Cr_2_Si_2_Te_6_, occurring over distances smaller than 0.6 nm, significantly narrows the band gap and enables stable semiconducting behavior down to 2 K. A dramatic increase in in-plane resistance occurs as the out-of-plane intrinsic FM transition emerges and strengthens with decreasing temperature below 50 K.

**Figure 6:**
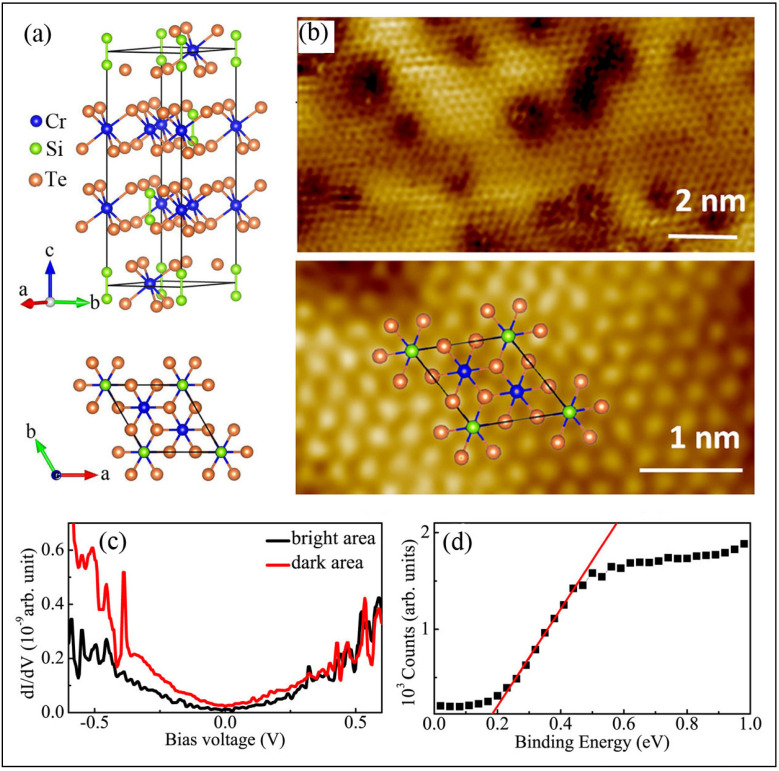
(**a**) Crystal structure of Cr_2_Si_2_Te_6_ (space group: R-3h) shown from side and top views. (**b**) STM images: upper image taken with a sample bias of 1 V and tunneling current of 0.8 nA; lower image taken with a sample bias of 1.3 V and tunneling current of 0.8 nA. Atoms are presented as follows: green for Si, blue for Cr, and brown for Te. (**c**) Tunneling spectroscopy comparison between the bright (defect-free) and dark (defective) regions. (**d**) UPS of Cr_2_Si_2_Te_6_ at room temperature. (**a**–**d**) are reprinted with permission from [130]. Copyright (2022) American Chemical Society.

As shown in Figure 6a, Cr_2_Si_2_Te_6_ is a layered material with Si−Si pairs. Cr atoms form a honeycomb lattice in planes separated by vdW bonds, with Si at the center of each hexagon. Each Cr is octahedrally coordinated by Te, and Si−Si bonds form dimers, creating Si_2_Te_6_ groups [174,175,176]. Magnetic interactions within planes are FM, arising from Cr−Te−Cr superexchange through a nearly 90° angle, and are stronger than the AFM Cr−Cr direct exchange. Cr_2_Si_2_Te_6_ exhibits FM order below 32 K and has a band gap of 0.4 eV [177,178]. FM correlations along the c-axis vanish above 50 K, but in-plane short-range FM correlations persist up to room temperature [179].

The surface of the Te atomic layer is easily exposed through crystal cleaving. Figure 6b shows the STM topography of a cleaved Cr_2_Si_2_Te_6_ crystal, revealing an ordered honeycomb lattice of bright spots. Notably, a significant number of darker domains (defects) are also observed (Figure 6b, upper panel). A similar defect pattern was observed in Cr_2_Ge_2_Te_6_, which is attributed to the replacement of Cr by Ge atoms [180]. As observed in large-scale STM images, long-range atomic order is evident on both bright and most dark surface domains, except for vacancy-like dark spots, which appear as random waviness or corrugation of the top atomic layers. This apparent waviness in the STM image could result from either geometric or electronic effects. Geometrically, random variations in the interlayer distance may lead to fluctuating interlayer electronic coupling in real space. In momentum space, this could result in changes in band dispersion within the Brillouin zone, affecting electron correlations. This behavior is akin to twisted or compressed bi- or multilayer graphene, where the interlayer coupling is dramatically altered, converting linearly dispersed Dirac cones into flat bands at magic twist angles [16] or under pressure. This can give rise to a correlated electronic liquid, which may exhibit skyrmions in the FM state or unconventional SC [16,181]. Electronically, local variations in the electronic structure could also contribute to the observed surface waviness. In either case, whether due to geometric or electronic effects, these observations suggest the presence of in-plane electronic heterostructures on the surface layers.

Figure 6c shows a comparison of the spatially averaged dI/dV spectrum of bright and dark areas measured at room temperature. A small energy gap around E_F_ is observed, which is narrower in the dark area. The bright and dark areas are separately interconnected, each forming electrical transport channels with different electronic band gaps. The average energy gap between the valence band maximum and the Fermi level is approximately 0.16 eV, as determined from the ultraviolet photoelectron spectroscopy (UPS) results in Figure 6d. The randomly distributed bright areas act as electron scattering centers for electrons in the dark channels, while the dark areas serve as electron trap centers for electrons in the bright channels. Both effects lead to Anderson localization, resulting in less dispersive and more correlated conducting bands.

**Figure 7:**
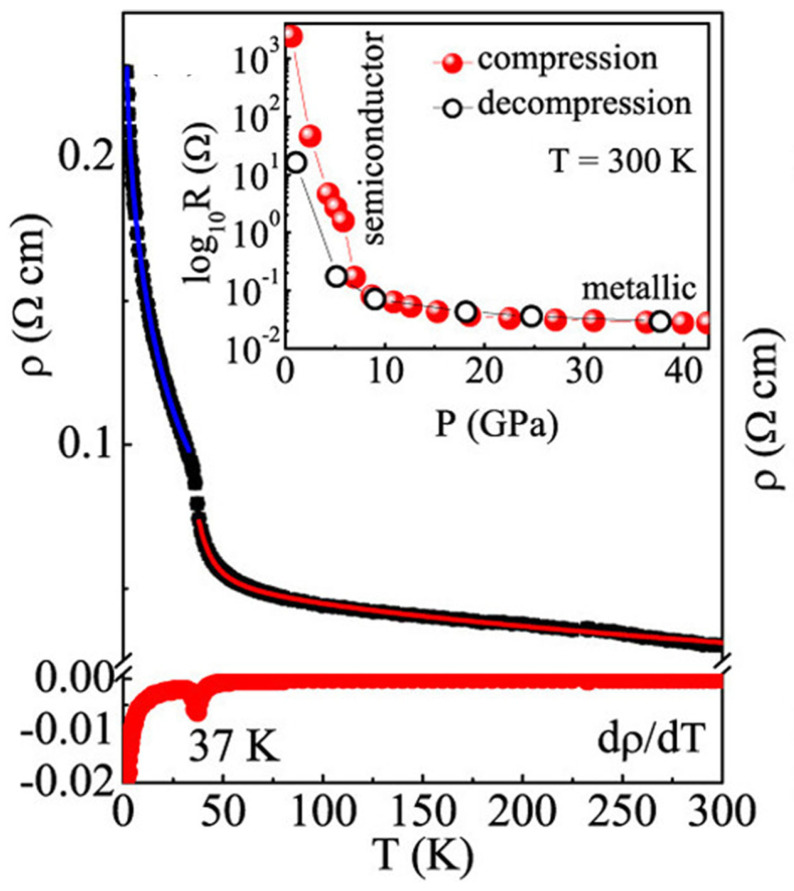
Temperature dependence of in-plane electrical resistivity, ρ(T) (**top**), and its derivative, dρ/dT (**bottom**), in zero-field mode for a Cr_2_Si_2_Te_6_ single crystal. The inset shows the room-temperature resistance of a bulk Cr_2_Si_2_Te_6_ sample during compression and decompression. Figure is reprinted with permission from [130]. Copyright (2022) American Chemical Society.

The resistivity ρ(T) of Cr_2_Si_2_Te_6_ at ambient pressure, shown in Figure 7, increases as the sample cools from room temperature, exhibiting typical semiconducting behavior. Below 50 K, ρ(T) rises more rapidly, with a weak shoulder around 37 K, coinciding with the FM transition temperature (T_c_), reflecting the effect of magnetization on resistance. Since the FM correlations along the c-axis emerge below 50 K [31], here, we should point out that the rapid increase in in-plane resistance below ~50 K could be attributed to carrier cyclotron motions induced by the emergence and enhancement of out-of-plane FM magnetization as temperature decreases. The cyclotron motions of neighboring carriers effectively cancel each other’s longitudinal electrical transport, leading to a dramatic increase in resistivity. Above 40 K, in the paramagnetic state, the resistivity follows a thermally activated model accounting for the disorder-induced localization of electronic wave function: ρ(T) = ρ_0_ + A exp(E_ρ_/k_B_T) − BT^0.5^. Here, ρ_0_ represents residual resistivity, while A and B are the prefactors for activated and localization scattering mechanisms, respectively, and E_ρ_ is the activation energy [182,183]. The fit yields E_ρ_ ≈ 18.0 meV, A = 10(2) × 10^−5^, and B = 1.77(3) × 10^−3^, indicating that electronic transport is dominated by disorder-induced localization.

Below the magnetic transition, the resistivity ρ(T) can be fitted using the same equation as in the paramagnetic state, but with band conduction replaced by adiabatic small polaron hopping. The resistivity is expressed as follows: ρ(T) = ρ_0_ + CT exp(E_ρ_/k_B_T) − DT^0.5^. Here, the temperature-activated term A exp(E_ρ_/k_B_T) is replaced by CT exp(E_ρ_/k_B_T) [184]. The derived values are E_ρ_ ≈ 0.30 meV, C = 2.3 × 10^−3^, and D = 43 × 10^−3^.

It should be noted that disorder-induced localization is dominant in both the paramagnetic state and below the magnetic transition, indicating a strong correlation between electronic transport and inhomogeneous nanoscale short-range crystallographic distortions.

Here, we experimentally observed that electrical transport is a sensitive probe for detecting magnetic order and disorder. Nanoscale defects in the 2D vdW interface reshape local bandgaps, creating spatially and electronically inhomogeneous conduction channels, which lead to Anderson localization and polaron-dominated hopping. Below the ferromagnetic transition, the emergence of out-of-plane magnetic order dramatically suppresses in-plane conductivity, which we attribute to magnetization-induced carrier cyclotron effects. The narrowed band gap induced by defects results in the emergence of a metallic state at high pressure, without any change in crystal lattice symmetry, as shown in the inset of Figure 7. This interplay critically shapes the temperature dependence of resistivity and demonstrates how 2D structural disorder and magnetic ordering together govern transport behavior.

When Cr_2_Si_2_Te_6_ is thinned below 28 nanometers, its electrical transport behavior changes significantly. Below the Curie temperature (33 K) and under a magnetic field perpendicular to the flakes, the magnetoresistance, which describes how resistance responds to a magnetic field, flips its sign [185]. In thick samples, magnetoresistance increases under a magnetic field due to dominant orbital scattering, while in thin flakes, magnetoresistance decreases as ferromagnetic interactions dominate, due to a reduced number of magnetic domains and weakened interlayer coupling. Additionally, when the magnetic field is applied parallel to the flake, the magnetoresistance remains uniformly negative across all temperatures and thicknesses, as the field aligns in-plane spins and suppresses spin fluctuations, spin disorder, and orbital scattering. The strongest negative magnetoresistanc is observed at low temperatures and in flakes of intermediate thickness of 28 nm, where ferromagnetic ordering is strong and orbital scattering is less pronounced [185]. These findings provide new insights into how sample thickness influences transport properties and spin correlations in vdW ferromagnets.

## 5. Future Directions

Future research could explore thickness-dependent transport and collective electron phenomena in single or few-layer 2D materials, alongside precise atomic-scale doping and defect control. Under ultra-high-vacuum conditions, constructing vertical and lateral heterostructures by exfoliating, stacking, and twisting 2D materials, and characterizing them to prevent oxidation and contamination, would be essential for advancing the understanding of idealized model systems. Developing advanced computational models will further support material and device optimization. Ultrafast spectroscopy, including time-resolved photoemission and transient absorption, could capture valence fluctuations in SC on femtosecond timescales and reveal their coupling to electronic, lattice, and spin dynamics under varying conditions [186,187,188,189,190]. Strain engineering in 2D materials could effectively modulate competing electronic phases and tune CDW states by altering lattice structures, wavevectors, transition temperatures, and electron–phonon coupling [191,192,193,194,195]. Another promising direction is to optimize the magnetization transition temperature and refine device engineering in 2D TMDs with high conductivity and strong spin–orbit coupling for spintronic applications [121,196,197,198].

## 6. Conclusions

We provide a comprehensive overview of transport properties across a broad range of 2D materials, with a focus on the interplay among intertwined coherent, incoherent, and disordered phases in collective electronic systems, considering electrical transport, CDW, SC, magnetic order, and defects—an important yet underexplored area of study. In particular, by employing a novel approach to modify quasi-2D vdW interfaces of layered structures, interdependence among these phases is observed, revealing quasi-2D characteristics in layered crystals as follows:

ZrTe_3_, featuring quasi-1D Te(2)-Te(3) lattice chains at the vdW interface, forms CDW below 63 K and SC below 2 K. Chemical doping with Se, Hf, Cu, or Ni modifies chain distances, influencing electron–phonon coupling and valence states, thereby tuning electrical transport properties and the onset temperatures of CDW and SC. Specifically, Cu or Ni intercalation in the vdW gap or the Se substitution of Te lattice atoms in quasi-1D atomic chains weakens electron–phonon coupling, suppressing CDW formation and enhancing SC. In contrast, the Hf substitution of Zr strengthens electron–phonon coupling, stabilizing the CDW phase and suppressing SC. These findings reveal the competitive relationship between CDW and SC, which can be tuned through foreign atom doping. Furthermore, through the additional and comprehensive analysis conducted herein, we provide new insight into ‘mixed bulk–filament superconductivity,’ and propose an unconventional SC mechanism, in which valence-state fluctuations at the vHS, driven by quasi-1D Te lattice vibrations, enable Cooper pair formation and transfer between the quasi-1D and 3D Fermi bands at the vHS, leading to unconventional SC.

For 2H-MxTaS_2_ (M = Mn, Co), the intercalation of M atoms in the vdW gap induces FM with easy-plane anisotropy in 2H-Mn_x_TaS_2_ and strong uniaxial out-of-plane anisotropy in 2H-Co_0.22_TaS_2_, which eventually evolves into three-dimensional AFM in 2H-Co_0.34_TaS_2_. The temperature-dependent resistivity of all samples shows metallic transport behavior, with clear resistivity kinks at magnetic transition temperatures in 2H-Mn_0.28_TaS_2_ and 2H-Co_0.34_TaS_2_, reflecting the coupling between magnetism and electrical transport.

Cr_2_Si_2_Te_6_ crystals exhibit magnetic ordering within a few atomic layers, with randomly distributed defects that possess a short-range crystal order, forming in-plane electronic heterostructures with a nested electronic band gap. This results in electronic conductivity with sensitivity to magnetic transitions that are dominated by polaron hopping. Additionally, the electronic conductivity is highly sensitive to magnetic transitions. The ability to narrow the electronic band gap through defect engineering opens new avenues for manipulating spins via electrical conduction.

The above demonstrated that foreign atom intercalation, lattice subtraction, and defect engineering in 2D vdW interfaces of layered crystals are capable of tuning their atomic, electronic, and vibrational structures, which, in turn, affects electrical transport and collective coherent electronic phenomena, such as CDWs, SC, and magnetism, offering insights into the intricate interdependencies and interplay among these phenomena, and holding potential for advancing next-generation electronic, spintronic, and quantum technologies.
